# Single horizontal Y-V vermilion plasty including orbicularis oris muscle repair for secondary correction of the whistling defect: A universal technique

**DOI:** 10.4317/medoral.18477

**Published:** 2012-12-10

**Authors:** Emeka Nkenke, Elefterios Vairaktaris, Florian Stelzle, Konstanze Scheller

**Affiliations:** 1 MD, DMD, M.A., PhD, Associate Professor, Department of Oral and Maxillofacial Surgery, Erlangen University Hospital, Erlangen, Germany; 2 MD, DMD, PhD, Professor and Head, Department of Oral and Maxillofacial Surgery, University of Athens Medical School, Attikon Hospital, Athens, Greece; 3 MD, DMD, PhD, Associate Professor, Department of Oral and Maxillofacial Surgery, Erlangen University Hospital, Erlangen, Germany; 4 MD, DMD, Assistant Professor, Department of Oral and Maxillofacial Surgery and Facial Plastic Surgery, Martin-Luther-University of Halle-Wittenberg, Germany, Halle, Germany

## Abstract

Objectives: The present prospective study aimed at objectively evaluating the relevance of a single horizontal Y-V vermilion plasty including orbicularis oris muscle repair for secondary correction of whistling deformities in unilateral as well as bilateral cleft lip cases. Study Design: Ten patients were included in the study (mean age 20.2±6.2 years). The size of the whistling defects was determined on photographs before and 12 months after surgery. Additional surgical procedures like columella lengthening and rhinoplasty were documented. Results: Seven minor and 3 moderate whistling defects were corrected. In 7 patients additional procedures were carried out. The data of the 12 months follow-up showed that the whistling defect was significantly reduced in size (p<0005). In 7 out of 10 patients the result of surgery was rated “good” and in 3 patients “moderate”. Conclusions: The present prospective study is the first one to show on an objective basis that the presented technique allows reducing whistling deformities significantly with good overall results in the majority of the cases. Moreover, the technique can be combined with other corrective procedures like columella lengthening without problems. As a consequence, it is a relevant and universal surgical technique for the correction of whistling defects.

** Key words:**Bilateral cleft lip, unilateral cleft lip, secondary correction, vermillion plasty, whistling defect.

## Introduction

The whistling deformity is a concave defect of the vermillion in the central portion of the upper lip. It is one of the most frequent sequelae of primary lip repair (1). The underlying causes of this defect are an intrinsic deficiency of tissue in the prolabium and the absence of continuity of the orbicularis oris muscle (2,3). Sometimes, the whistling deformity is even accentuated in bilateral cleft lip patients because improperly attached muscles produce a bulge in the lateral lip segments (4,5).

As a consequence, a number of authors have stressed the importance of orbicularis oris muscle repair in primary cleft lip surgery in order to avoid the whistling deformity (2,6). The relevance of muscle repair has also been emphasised for the correction of secondary deformities (3,5). Restoring the continuity of the orbicularis oris muscle is considered the basis for a successful and permanent correction (1,3). Although successful correction of the whistling defect also has been achieved without touching the orbicularis oris muscle (7), it has been stressed that an isolated revision of scars of the lip often will be followed by recurrence of the deformity (3).

A number of different methods for secondary repair of the whistling defect have been described in the current literature (1-4,7-16). They are compiled in  Table 1. Some of these techniques are technically demanding and none have been found univer-sally applicable for all whistling deformities (7). So far, the selection of a specific technique for the correction of whistling deformities is based on personal preferences. Prospective studies that increase the level of evidence in the field are missing. Therefore, the present prospective study aimed at evaluating the relevance of a single horizontal Y-V vermilion plasty including orbicularis oris muscle repair for secondary correction of whistling deformities in unilateral as well as bilateral cleft lip cases.

**Table 1:**
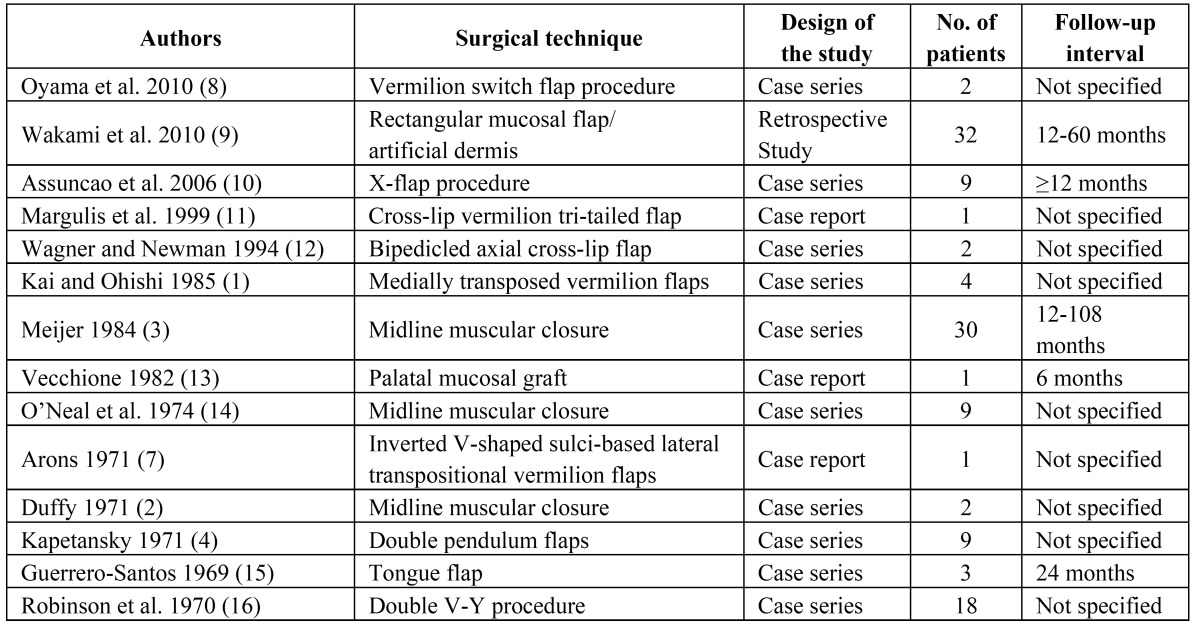
Studies highlighting different techniques of secondary correction of the whistling deformity.

## Material and Methods

The prospective study was approved by the ethics committee of the University of Erlangen-Nuremberg. Type of cleft, gender and patient age at the time of surgery were documented. Patients were included in the study when primary closure of the lip had been carried out within the first year of life and no frontal teeth of the maxilla as well as of the mandible were missing. Patients were excluded from the study if they had undergone previous attempts to correct for the whistling deformity.

**Figure 1:**
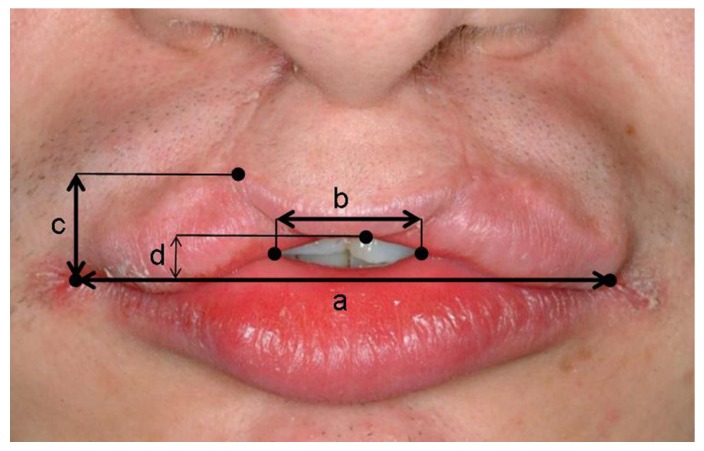
Outline of the distances needed for the calculation of the defect score. The whistling deformity belongs to patient no. 1 ( Table 2 and  Table 3, preoperative defect score 769). a: Intercommisural distance (distance between left and right oral commissure) b: Maximum defect width (distance between the most lateral left and right points of the whistling deformity measured on a line parallel to the intercommisural line) c: Maximum height of the upper lip (distance between the intercommisural line and the most cranial point of the vermilion measured on a line perpendicular to the intercommisural line) d: Maximum defect height (distance measured on a line perpendicular to the intercommisural line and the most cranial point of the whistling deformity).

Defect ratios were assessed for the whistling deformities of the patients on frontal photographs (9). The horizontal defect ratio was calculated as “maximum defect width / intercommisural distance x 100” (Fig. 1). The vertical defect ratio was calculated as “maximum defect height / maximum height of the upper lip x 100”. The product of both ratios was defined as the defect score. Measurements were made from frontal photographs at rest. The whistling deformity was classified as “minor” defect when the defect score was below 400, “moderate” defect when the score was between 400 and 1400 and as “severe” defect when the score was above 1400. Measurements were carried out on preoperative photographs and photographs taken 12 months after surgery.

When the correction of the whistling defect was accompanied by additional operations (e.g. columella lengthening or rhinoplasty) these procedures were documented. During the initial 10 days of the postoperative course complications like wound breakdown, haematoma and infection were documented.

Twelve months after surgery the results of the whistling defect correction were again evaluated on photographs (9). The outcome was rated “good” when there was no sagittal vermilion deficiency and a well filled vermilion tubercle. The outcome was “moderate” when the sagittal vermilion deficiency was resolved in a way that the anterior teeth were hidden but the vermilion tubercle was unclear or flat. A “poor” outcome showed persisting sagittal vermilion deficiency as well as vermilion tubercle deficiency.

-Surgical technique

**Figure 2:**
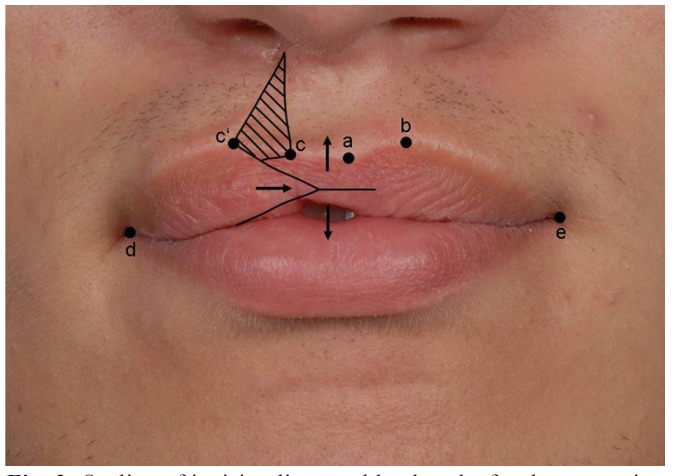
Outline of incision lines and landmarks for the correction of the whistling deformity in patient no. 2 ( Table 2 and  Table 3, preoperative defect score 355). a: Most caudal point of Cupid’s bow b: Most cranial point of Cupid’s bow on the non-cleft side c: Most cranial point of Cupid’s bow on the cleft side c’: Point corresponding with c d: Right commissure e: Left commisure Hatched area: Cutaneous tissue to be excised in order to correct for the unnaturally wide philtrum Horizontal arrow: Direction of advancement of the lateral triangle Vertical arrows: Directions of movement of prolabium and oral portion of the upper lip.

**Figure 3:**
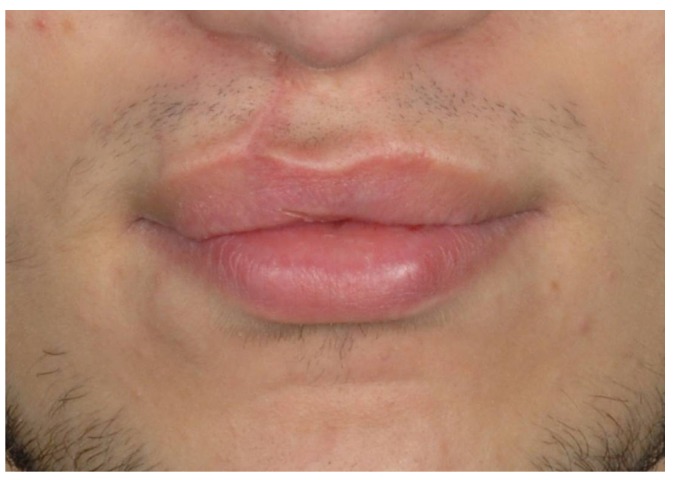
Upper lip of patient no. 2 12 months after surgery (defect score 0, outcome was rated “good”).

Surgery was carried out under general anaesthesia. All operations were performed by the same surgeon (E.N.). The surgical technique was the same for unilateral as well as bilateral cleft lip cases (Figs. 2,3). An initial horizontal incision was made along the center of the whistling deformity extending over one of the lateral edges of the defect. The depth of the incision included the full thickness of the orbicularis oris muscle. Subsequently, a horizontal V-incision was carried out on the opposite aspect of the defect creating a lateral triangular flap. The facial aspect of the V-incision extended to the cutaneo-vermilion border. The oral aspect of the V-incision was performed mirror-inverted. Again the depth of the incisions included the orbicularis oris muscle.

The orbicularis oris muscle bundles on both sides of the deformity were dissected from the subcutaneous tissue and freed up to the nasolabial line. The muscle of the V-shaped part was advanced medially towards the contralateral side. By lateral mobilization and midline suturing of the corresponding muscle segments the continuity of the orbicularis oris muscle was restored and at the same time the bulging of the lateral upper lip was corrected. The muscle parts were sutured together with slowly resorbable material (PDS II 4-0, Ethicon, Norderstedt, Germany). At the level of the nasal spine both bundles were sutured to the dense fibrous tissue at the base of the spine.

Subsequently, also the lateral vermilion triangular flap was moved centrally until it reached the end of the incision on the contralateral side. As a consequence, the tissue borrowed from the lateral side added to the central fullness of the vermilion, reconstructed the vermilion tubercle and protruded the prolabium. The vermilion incisions were closed with resorbable sutures (Vicryl 5-0, Ethicon, Norderstedt, Germany).

When an unnaturally wide philtrum was present, a vertical incision of the skin was made from the nostril floor downward to the vermilion. The depth of this incision was limited to the subcutaneous tissue. The excision of skin of the white lip included the excessive width of prolabium skin in a triangular fashion with the basis of the triangle at the cutaneo-vermilion border. The cutaneous incisions were closed with resorbable sutures (Monocryl 6-0, Ethicon, Norderstedt, Germany).

-Statistics

Mean values are given with standard deviations. For comparison of continuous variables in paired samples, the Wilcoxon test was used. P-values?05 were considered significant. The measurements of the defect scores were repeated after 4 weeks in order to assess the intrarater reliability by calculating Cohen’s kappa. All calculations were made using SPSS Version 14.0 for Windows (SPSS, Chicago, USA).

## Results

Ten patients were included in the study. All of them were males. The patients had a mean age of 20.2±6.2 years ( Table 2). The cleft lip deformity was unilateral in 8 cases and bilateral in 2 cases.

**Table 2:**
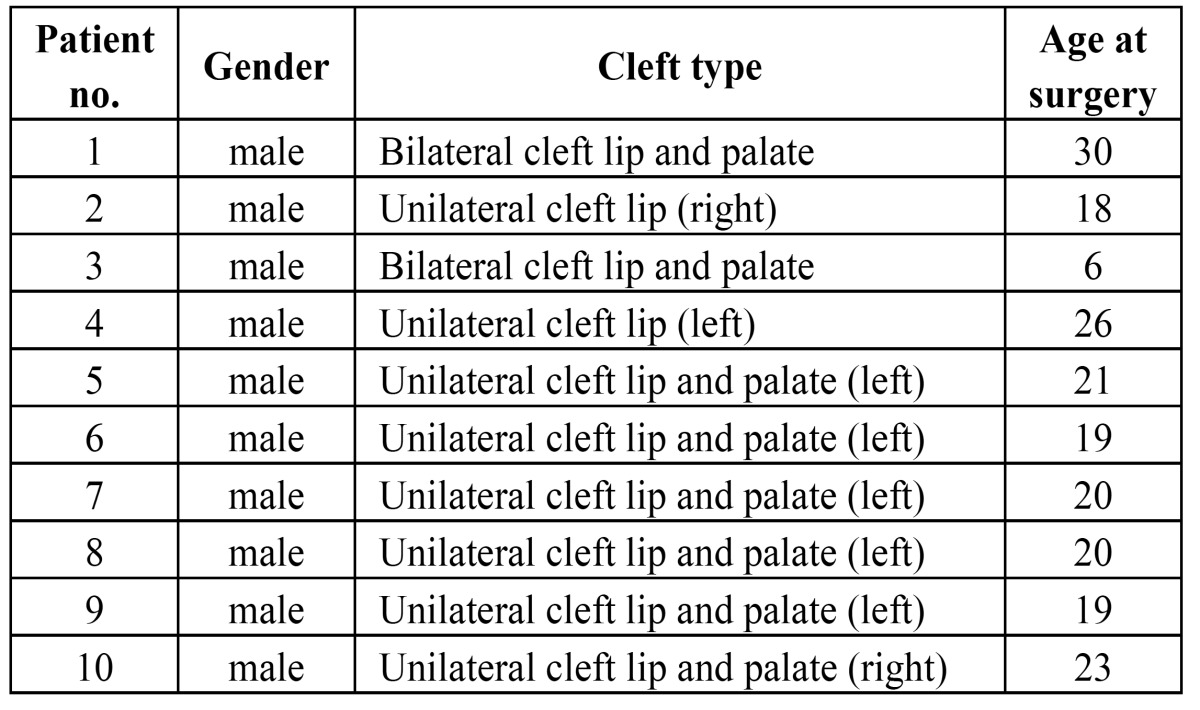
Basic demographic data of the patients undergoing secondary repair of a whistling defect (mean age 20.2±6.2 years).

Preoperatively, 7 patients showed a minor whistling defect (<400) and 3 patients a moderate whistling defect (400-1400 points,  Table 3). There were no patients with a severe defect. The mean value of the preoperative defect score was 399.5±215.9. After 12 postoperative months the defect score was 18.0±25.9. Compared to the preoperative situation it was significantly reduced (p<0005). The Cohen`s kappa value revealed a high intrarater reliability with a significant correlation between the measurements taken at two different points of time (p<0005).

**Table 3:**
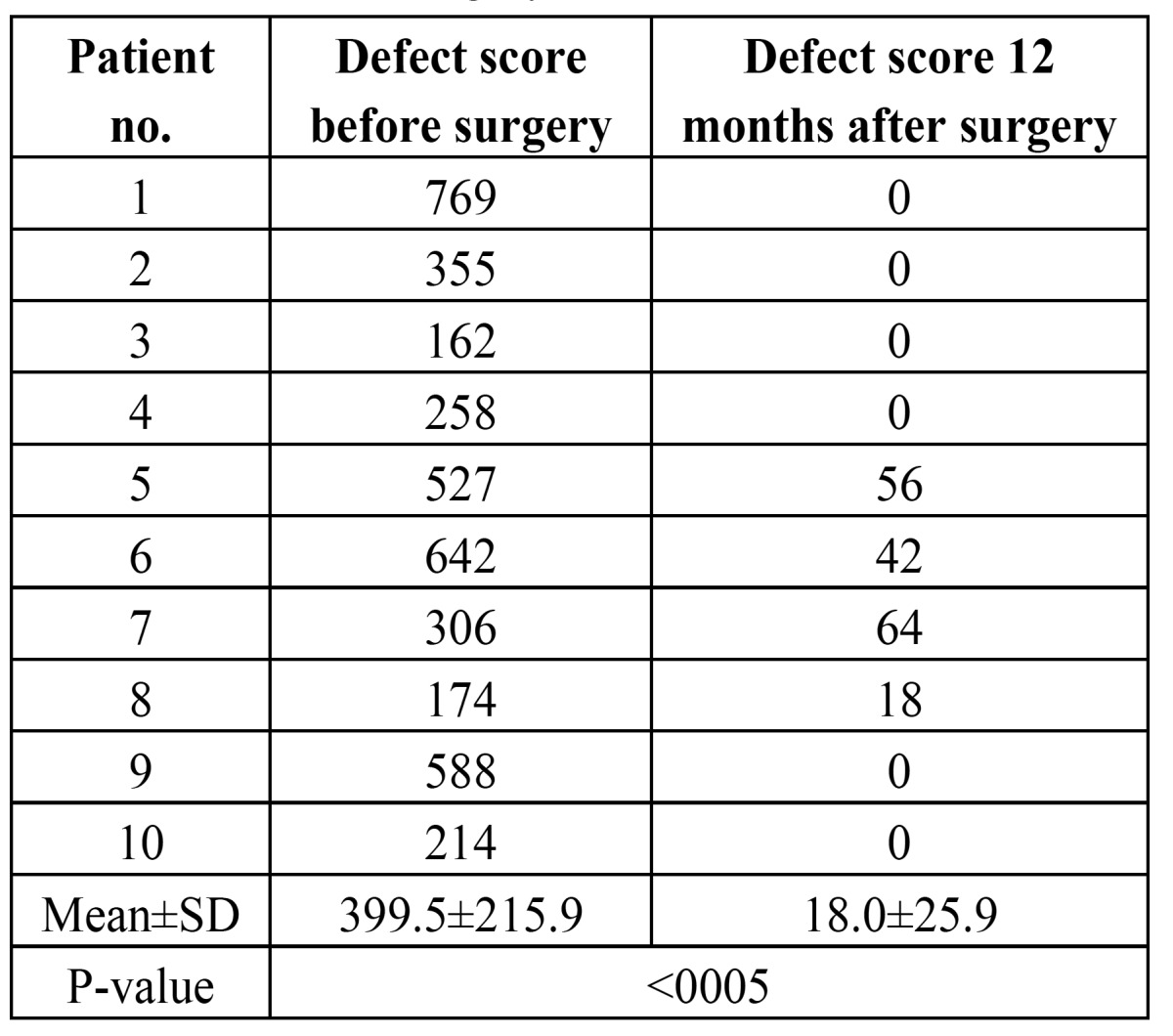
Defect score of the whistling deformity before and 12 months after surgery.

In 7 patients additional procedures (3 times rhinoplasty, 2 times columella lengthening, 2 times a combination of rhinoplasty and columella lengthening) were carried out in addition to the None of the patients showed wound breakdown, haematoma of infection during the postoperative course.

Twelve months after surgery the outcome of correction of the whistling defects was rated “good” in 7 cases, “moderate” in 3 cases and “poor” in none of the cases.

## Discussion

A number of different techniques have been described for secondary repair of the whistling defect ( Table 1). All procedures have in common that they are directed to supplying tissues to fill in the deficit of the vermillion. However, some authors do not pay attention to the repair of the orbicularis oris muscle (7,13,15,16), while others question the long-term success of procedures that do not reconstruct the continuity of this muscle repair when correcting a whistling defect. To date, there are no clear criteria for the selection of one or the other technique. Prospective studies that increase the level of evidence in the field are missing. So far, prospective studies on the topic are missing that allow increasing the level of evidence in the field. Therefore, the present prospective study aimed at evaluating the relevance of a single horizontal Y-V vermilion plasty including orbicularis oris muscle repair for secondary correction of whistling deformities in unilateral as well as bilateral cleft lip cases.

A double Y-V procedure has been described previously (16). The authors put an emphasis on the transposition of mucosa in the whistling defect in order to correct for the missing volume. However, it seems that they did not perform a repair of the orbicularis oris muscle. Neglecting muscle reconstruction might have been the reason for the adoption of a second V-flap in order to correct adequately for the volume deficiency. The results of the present study seem to show that when the muscle segments are dissected and the orbicularis oris muscle continuity is restored, there is no longer a need for a second V-flap even in bilateral cleft lip cases. As a consequence, as far as the incision pattern is concerned the surgical technique becomes more straightforward.

In the past, authors have debated about the question if the correction of a whistling defect and the concomitant repair of the orbicularis oris muscle should keep the white lip intact. Different methods have been developed that allowed not touching the cutaneous aspect of the upper lip. For example a procedure has been described in which two vertical pendulum flaps composed of vermillion and orbicularis oris muscle are swung medially to fill in the whistling defect (4). On the other hand, it has been criticised that most whistling defect cases also need revision of the white lip because of scars, an unnaturally wide prolabium, a short columella or a wide nostril floors. Therefore, it appeared to some authors that avoiding an incision of the white lip does not bear advantages (1,6). The surgical technique described in the present study does not necessarily require an incision of the white lip. However, additional procedures like columella lengthening or correction of the width of the prolabium could not be carried out without incisions of the white lip. Therefore, not touching the white lip was not seen as a major aim in the present study. Instead, surgery to the white lip seemed to help to improve the outcome of the operations. These findings are in line with previous reports (1,3,5,6).

Seven out of 10 patients in the present study required additional procedures beside the correction of the whistling defect. Therefore, it seems that a technique for the repair of the whistling defect should allow correcting for other aspects simultaneously. The technique described in the present study does not hinder carrying out additional procedures. This seems to be a major advantage compared to other methods that for example cannot be combined with columella lengthening (3,17).

Attempts have been made previously to objectively assess the size of whistling defects (9). The method has been adopted in the present study. A postoperative objective evaluation of the outcome of surgery is difficult to find in the current literature. To the best of our knowledge, the present study is the first one to prospectively assess the size of whistling defects before and 12 months after surgery. Future studies should adopted comparable methods for evaluation the size of whistling defects before and after surgery in order to allow comparing different methods for the correction of whistling defects.

The present study is limited by the fact that only minor and moderate whistling defects were operated on. However, the results of the study show that it was possible to significantly reduce the size of the defects with good overall results in the majority of the cases. With this positive outcome attempts will be made in the future to even establish the presented technique for major whistling deformities.

## Conclusions

The present prospective study is the first one to show on an objective basis that a single horizontal Y-V vermilion plasty including orbicularis oris muscle repair for secondary correction of whistling deformities leads to significant defect reduction in minor and moderate defects. In the majority of the cases the overall result was rated good. The technique can be combined with other corrective procedures like columella lengthening without problems.
